# Identification of a Bacteria-Like Ferrochelatase in *Strongyloides venezuelensis*, an Animal Parasitic Nematode

**DOI:** 10.1371/journal.pone.0058458

**Published:** 2013-03-13

**Authors:** Eiji Nagayasu, Sohta A. Ishikawa, Shigeru Taketani, Gunimala Chakraborty, Ayako Yoshida, Yuji Inagaki, Haruhiko Maruyama

**Affiliations:** 1 Department of Infectious Diseases, Division of Parasitology, Faculty of Medicine, University of Miyazaki, Miyazaki, Japan; 2 Graduate School for Life and Environmental Sciences, University of Tsukuba, Tsukuba, Japan; 3 Department of Biotechnology, Kyoto Institute of Technology, Kyoto, Japan; University of Melbourne, Australia

## Abstract

Heme is an essential molecule for vast majority of organisms serving as a prosthetic group for various hemoproteins. Although most organisms synthesize heme from 5-aminolevulinic acid through a conserved heme biosynthetic pathway composed of seven consecutive enzymatic reactions, nematodes are known to be natural heme auxotrophs. The completely sequenced *Caenorhabditis elegans* genome, for example, lacks all seven genes for heme biosynthesis. However, genome/transcriptome sequencing of *Strongyloides venezuelensis*, an important model nematode species for studying human strongyloidiasis, indicated the presence of a gene for ferrochelatase (FeCH), which catalyzes the terminal step of heme biosynthesis, whereas the other six heme biosynthesis genes are apparently missing. Phylogenetic analyses indicated that nematode FeCH genes, including that of *S. venezuelensis* (SvFeCH) have a fundamentally different evolutionally origin from the FeCH genes of non-nematode metazoa. Although all non-nematode metazoan FeCH genes appear to be inherited vertically from an ancestral opisthokont, nematode FeCH may have been acquired from an alpha-proteobacterium, horizontally. The identified *Sv*FeCH sequence was found to function as FeCH as expected based on both *in vitro* chelatase assays using recombinant *Sv*FeCH and *in vivo* complementation experiments using an FeCH-deficient strain of Escherichia coli. Messenger RNA expression levels during the *S. venezuelensis* lifecycle were examined by real-time RT-PCR. SvFeCH mRNA was expressed at all the stages examined with a marked reduction at the infective third-stage larvae. Our study demonstrates the presence of a bacteria-like FeCH gene in the *S. venezuelensis* genome. It appeared that *S. venezuelensis* and some other animal parasitic nematodes reacquired the once-lost FeCH gene. Although the underlying evolutionary pressures that necessitated this reacquisition remain to be investigated, it is interesting that the presence of FeCH genes in the absence of other heme biosynthesis genes has been reported only for animal pathogens, and this finding may be related to nutritional availability in animal hosts.

## Introduction

Heme is essential for the vast majority of life serving as a prosthetic group for many hemoproteins such as catalase, cytochrome, hemoglobin, myoglobin, and peroxidase [1]. Although most aerobic organisms possess a complete biosynthetic pathway for this compound [2], certain organisms are deficient in heme biosynthesis, lacking some or all genes for the hemebiosynthetic pathway. Some anaerobic protists, such as *Giardia intestinalis*, *Trichomonas vaginalis*, *Entamoeba histolytica*, *Cryptosporidium parvum*, *Blastocystis hominis*, and *Encephalitozoon cuniculi* do not possess any heme biosynthetic genes [3]. Members of the family Trypanosomatidae lost some or the entire set of heme biosynthesis genes. They acquire heme or heme precursors from their diet [3], [4]. In Trypanosomatidae, members of the genus *Trypanosoma* lack all the heme biosynthesis genes [3], [5], [6], [7], whereas other members such as *Leishmania* spp. possess the genes for the last three steps which were horizontally acquired from a gamma–proteobacterium [3]. Insect trypanosomatid species (*Blastocrithidia culicis* and *Crithidia oncopelti*) cannot synthesize heme by themselves but harbor bacterial endosymbionts that generate and donate heme or heme precursors to the host (trypanosomatid) cells [4], [8]. More peculiar is the case of *Phytomonas serpens*, a plant kinetoplastid [9]. This organism lacks most of the known hemoproteins including respiratory cytochromes and does not require heme for viability despite its dependence on oxidative metabolism [9]. The draft genome of *P. serpens* does not appear to contain heme biosynthesis genes other than ferrochelatase (FeCH, EC 4.99.1.1) [9].

Another important and interesting group of organisms that lack the ability to synthesize heme is the nematodes. Nematodes, or roundworms, are typically small, diverse, and highly abundant metazoan organisms [10]. Although free-living species are found in nearly all habitats (marine, freshwater, and soil), nematodes are also parasites of vertebrate and invertebrate animals as well as plants. Molecular phylogenetics have defined five major nematode clades (I through V), within which parasitism has arisen multiple times [11]. The genome of *Caenorhabditis elegans*, which was the first metazoan genome to be completely sequenced [12], appears to lack all seven genes necessary to synthesize heme from 5-aminolevulinic acid [13].

Some hemoproteins of animal parasitic nematodes are particularly well studied because of the interests in their roles in low-oxygen environment (host intestine). One such protein is perienteric hemoglobin of *Ascaris lumbricoides* (parasitic nematode of humans), which has an extraordinary high oxygen affinity, approximately 10,000-fold higher than that of the host’s globin [14]. The proposed functions of this oxygen-avid hemoglobin include oxygen detoxification by a reaction driven by nitric oxide [15] and maintenance of body wall O_2_ tension by creating an inward-decreasing O_2_ gradient that is considered important for oxygen unloading from body wall myoglobin, another heme-containing protein [16]. Another example of well-studied nematode hemoproteins is cytochrome *b* in the mitochondrial respiratory complex II of *Ascaris suum* (swine parasitic nematode). *A. suum* larvae utilize classic mammalian-type respiration, expressing a small subunit of larval cytochrome *b* (CybS^L^) [17]. In contrast, adult worms live in the host small intestine, where oxygen tension is low and utilize an anaerobic NADH-fumarate reductase system expressing a different small subunit of cytochrome *b* (CybS^A^) instead of CybS^L^ [17]. Given the important roles played by the hemoproteins in animal parasitic nematodes, it is interesting to know how heme molecules are synthesized or acquired from the animal hosts.

*Strongyloides* is a genus of obligate gastrointestinal parasites of vertebrates that belong to nematode clade IV [18]. Among more than 50 documented species, two are known to cause human infections, namely *Strongyloides stercoralis* and *Strongyloides fuelleborni* [18]. It is estimated that 30–100 million individuals are infected with *Strongyloides* worldwide primarily in tropic and subtropic regions [19]. Symptoms are usually absent or mild in immunocompetent hosts. However, in impaired host immunity, severe manifestations can develop, and fatalities may ensue [20].

To study strongyloidiasis, *Strongyloides venezuelensis*, which is native to rats but can also infect mice, has been widely used as a model [21]. In a transcriptome sequencing project of this *Strongyloides* species, we identified a partial cDNA sequence that most likely encodes a gene for FeCH [22]. FeCH catalyzes the terminal step of heme biosynthesis [23]. The existence of FeCH sequences was noticed in the genomes of *Brugia malayi* (another animal parasitic nematode belonging to nematode clade III) and its bacterial endosymbiont, (*Wolbachia*). However, further analysis was conducted only on the FeCH gene in the endosymbiont genome [24], [25].

In the present study, we cloned the entire cDNA sequence of the FeCH gene from *S. venezuelensis* (*Sv*FeCH). Our BLAST search on publically available databases revealed that only a fraction of nematode species possesses the FeCH gene. Interestingly, all these species were parasites of mammals. Surprisingly, in our phylogenetic analysis, nematode FeCH formed a distinctive clade, and it was placed distantly from the clade that contains non-nematode metazoan FeCH, suggesting that the origin of nematode FeCH genes are different from those of non-nematode metazoan FeCH. The chelatase activity of the *Sv*FeCH was confirmed by an *in vitro* assay using recombinant protein and a gene complementation assay using an FeCH-deficient *Escherichia. coli*.

Nematode genes for heme biosynthesis have not been cloned or characterized to date, essentially because of the nonexistence of these genes in species commonly used in laboratories such as *C. elegans*. Thus, the present study represents the first report of a cloned active FeCH from organisms in the phylum *Nematoda*.

Although the biological significance of carrying only the FeCH gene among other heme biosynthesis genes is unclear, the presence of this gene only in animal parasites suggests a possible role for this gene in nutritional adaptation to the animal host environment.

## Materials and Methods

### Ethics Statement

*S. venezuelensis* has been maintained over serial passages in male Wistar rats purchased from Kyudo Co. Ltd. (Kumamoto, Japan). The animals were housed and handled in the Division of Parasitology, Department of Infectious Diseases, University of Miyazaki [26]. All animal studies were conducted under the applicable laws and guidelines for the care and use of laboratory animals in the University of Miyazaki and approved by the Animal Experiment Committee of the University, as specified in the Fundamental Guidelines for Proper Conduct of Animal Experiment and Related Activities in Academic Research Institutions under the jurisdiction of the Ministry of Education, Culture, Sports, Science and Technology, Japan, 2006.

### 5′- and 3′-rapid Amplification of cDNA Ends (RACE)

To determine the sequences of the 3′- and 5′-ends of FeCH cDNA, RACE experiments were performed [27], [28]. The priming sites used for these experiments were based on a contig sequence obtained from our *S. venezuelensis* transcriptome sequencing project [22]. For 3′-RACE, a PrimeScript RT-PCR kit (Takara, Japan) was used with oligo(dT) adaptor primers to synthesize cDNA from total RNA prepared from parasitic adult worms. Using this 3′-RACE-ready cDNA as a template, hemi-nested PCR was performed first with primer pairs ENM059/ENM008, followed by ENM060/ENM008. The primer sequences used in this study are summarized in Table S1. The resultant PCR products were cloned into pCR2.1 TOPO (Invitrogen, Carlsbad, CA, USA) for DNA sequencing.

For 5′-RACE, a gene specific-primer (reverse) ENM070 was used to synthesize cDNA from total RNA prepared from adult worms. The addition of a homopolymeric A-tail to the 3′-end of the synthesized first-strand cDNA was performed using dATP and terminal transferase. The dA-tailed cDNA was used as a template for hemi-nested PCR first with primers ENM5_6_7, and ENM008/ENM071, then with primers ENM008/ENM072. The resultant PCR products were cloned into pCR2.1 TOPO for DNA sequencing.

Based on the sequence information obtained from the 5′- and 3′-RACE experiments, a PCR primer pair (ENM073/ENM074) was designed to amplify the entire ORF of the *Sv*FeCH gene. The PCR products obtained using an adult-stage cDNA sample as a template were cloned into pCR2.1 TOPO vectors to determine the sequence. The resultant full-length ORF sequence was deposited into DNA Data Bank of Japan under the accession number AB710465, which can be accessed through GenBank (http://www.ncbi.nlm.nih.gov/genbank/), and used to deduce the amino acid sequence of the *Sv*FeCH.

### BLAST Homology Search

To search for heme biosynthesis genes, BLAST homology searches [29] were performed against predicted protein sequence data from published nematode genome projects (*Caenorhabditis briggsae* (nematode clade V) [30], *C. elegans* (V) [12], Pristionchus pacificus (V) [31], Meloidogyne incognita (IV) [31], Meloidogyne hapla (IV) [32], *Bursaphelenchus* xylophilus (IV) [33], B. malayi (III) [25], Ascaris suum (III) [34], and Trichinella spiralis (I) [35]) and nematode expressed sequence tags (ESTs) from NEMBASE4 [36], and *S. venezuelensis* genome (obtained by the Roche-454 pyrosequencing platform [37] with an estimated coverage of more than 20, unpublished), and transcriptome [22] datasets, using human sequences as queries with cutoff value of 1×10^−4^. For the FeCH gene, the *S. venezuelensis* protein sequence, deduced from the cDNA sequence, was also used as a query to search for potential orthologs against the aforementioned set of nematode genome and EST datasets, as well as the NCBI nonredundant protein database. Similarly, nematode heme biosynthesis gene sequences identified during these database searches were used as queries, instead of the human sequences, to search for potential orthologs in our S. venezuelensis genome and transcriptome datasets.

### Phylogenetic Analyses

We retrieved the gene sequences encoding FeCH of 71 bacterial and 65 eukaryotic species from the GENBANK nonredundant protein database (note that some eukaryotes possess more than two FeCH homologs). These amino acid sequences and those of the *S. venezuelensis* homolog were firstly aligned using MAFFT [38], and the resultant alignment was edited manually. After the exclusion of ambiguously aligned positions, the final FeCH alignment containing 71 eukaryotic and 71 bacterial homologs with 177 amino acid positions was subjected to phylogenetic analyses, as described below. Taxonomic affiliation and accession numbers for the sequences considered in our FeCH alignment are listed in Table S2.

Maximum likelihood (ML) phylogenetic analyses were performed using RAxML 7.2.8 [39]. The substitution model used was the LG model incorporating the among-site rate variation approximated with a discrete gamma distribution with four categories (LG+Γ). This particular substitution model was selected as the most appropriate model for the FeCH alignment using Aminosan [40]. The ML tree was selected from heuristic tree search initiated from 20 distinctive parsimonious trees. In ML bootstrap analysis (with 100 replicates), a single tree search was performed per replicate.

Bayesian analysis based on the LG+Γ model was also conducted using MrBayes 3.2.1 [41]. Four parallel Metropolis-coupled Markov chain Monte Carlo runs, each consisting of one cold and seven heated chains with a chain temperature of 0.1, were run for 5,000,000 generations. Log-likelihood scores and trees with branch lengths were sampled every 1000 generations. The first 1,250,000 generations were excluded as burn-in, and the remaining trees were summarized to obtain Bayesian posterior probabilities.

### Bacterial Expression of Recombinant *Sv*FeCH and Measurement of Porphyrin-metal Chelatase Activity

A cDNA sequence corresponding to the entire catalytic core region of *Sv*FeCH (amino acid positions 29–373) was obtained by PCR using the primer pair TKT001/TKT002. The PCR product was cloned into pET-21a (+), an *E. coli* expression vector (Merck, Darmstadt, Germany), and the plasmid obtained was transferred to *E. coli* BL21. The bacteria were grown in LB medium for 16 h, and then the culture medium was diluted by 10-fold in fresh LB medium. The enzyme was expressed with 0.3 mM isopropyl-β-d-thiogalactopyranoside (IPTG) at 30°C for 2 h.

The cells were harvested by centrifugation and suspended in 20 mM Tris-HCl (pH 8.0), 10% glycerol, 1 mM DTT, 0.1% Tween 20, and 0.3 M NaCl. Cells were disrupted by sonication and centrifuged at 5000×g at 4°C for 10 min. The supernatants were used for the enzyme assay.

The FeCH activity was determined by measuring the insertion of zinc ions into mesoporphyrin, as described previously [42]. After incubation at 30°C for 30 min, the protoporphyrin or zinc-protoporphyrin formed was measured fluorophotometrically.

### Genetic Complementation Assay of hemH (Bacterial FeCH) Deficient *E. coli*

*E. coli* strain VS200 (ΔhemH), a deletion mutant for hemH gene [43] was provided by the National Bioresource Project of MEXT, Japan.

The entire ORF of *Sv*FeCH, obtained by RT-PCR with the primer pair ENM089/ENM098, was cloned into the *Xho*I/*Bgl*II restriction site of pFLAG-CTC plasmid, an *E. coli* expression vector containing a tac promoter (Sigma-Aldrich, St. Louis, MO, USA). The resultant plasmid pFLAG-CTC-*Sv*FeCH was tested as a gene complementation vector. The original pFLAG-CTC plasmid served as a control.

ΔhemH was transformed with pFLAG-CTC-*Sv*FeCH or with pFLAG-CTC. The transformed and untransformed *E. coli* ΔhemH strains were cultured overnight in LB medium supplemented with hemin (10 µg/ml).

For the culture of the transformed ΔhemH, ampicillin was also added at a concentration of 50 µg/ml. The bacteria from the overnight culture were pelleted by centrifugation and washed thrice with LB medium. After washing, the bacteria pellets were resuspended to give an OD_600_ of 0.1 in hemin-containing (10 µg/ml) or hemin-free LB medium with (for the transformed ΔhemH) or without (for the untransformed ΔhemH) ampicillin, and incubated at 37°C with rocking. O.D. 600 of each culture was measured every hour up to 20 h.

### Real-time RT-PCR Analysis

Total RNA samples were prepared from eggs, a mixture of first- and second-stage larvae (L1/L2), third-stage infective larvae (L3i), lung third-stage larvae (LL3), mucosal larvae (ML) and parasitic adult stages. Eggs were obtained by the floatation method with saturated salt solution from rat feces. L1/L2 and L3i were prepared from fecal culture. LL3 and ML were collected from infected male ICR mice 72 and 85 h after infection, respectively. Parasitic adults were collected from the small intestine of rats 10 days after infection. Eggs and worms were washed extensively with PBS, pelleted by centrifugation and stored at −80°C until used.

Frozen eggs or worms were crushed with a crushing device (SK-200) purchased from Tokken, Japan. Trizol (Invitrogen) was used for total RNA preparation following the manufacturer’s instructions. After DNase I treatment, cDNA was synthesized using PrimeScript RT-PCR kit. Real-time RT-PCR was performed by the GoTaq qPCR system (***Promega***, Madison, WI, USA) using specific primer pairs (ENM056/ENM057 for *Sv*FeCH and 377F/501R for 18S ribosomal RNA genes). The real-time RT-PCR analyses were performed using biological triplicate samples.

## Results

**Figure 1:**
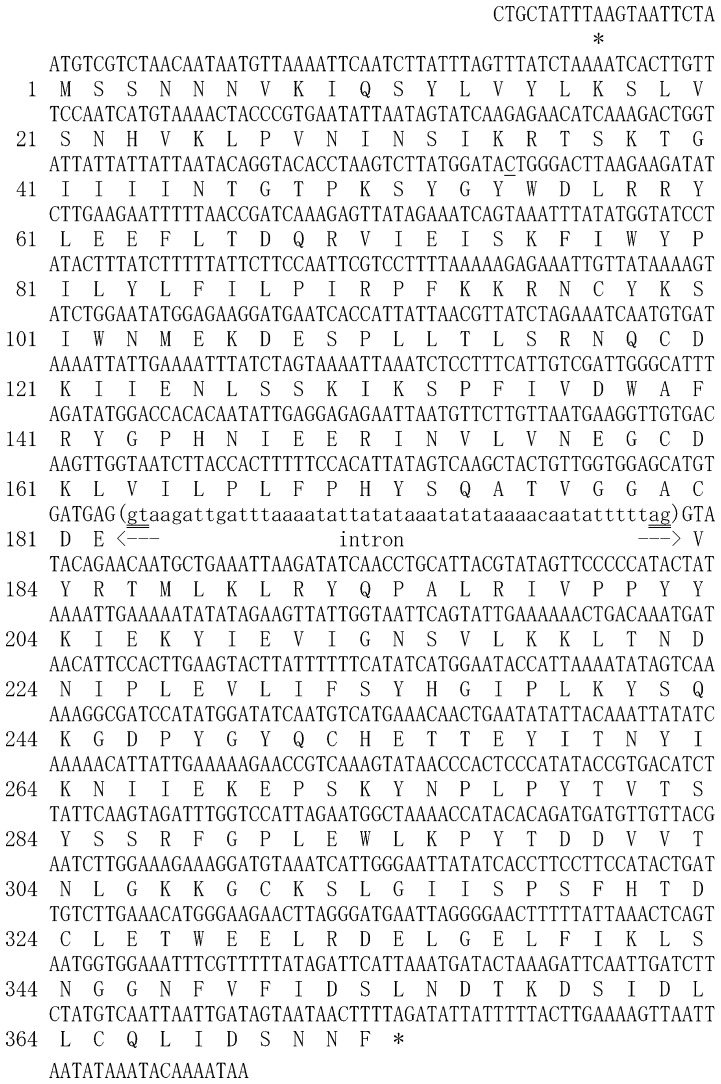
Genomic DNA and cDNA sequences of the *Strongyloides venezuelensis* FeCH gene. Both sequences were identical excluding the intronic region, which existed only in the genomic DNA, and a nucleotide at the 54th codon (single-underlined), which was cytosine in the cDNA sequence but was thymidine in the genomic DNA sequences (silent mutation). The deduced amino acid sequence is shown below the nucleotide sequence. In-frame stop codons are indicated by asterisks. 5′- and 3′-splice junction sites that obey the GT-AG rule of eukaryotic introns are indicated by double lines.

Initially, we identified an EST contig that appeared to represent a transcript from *Sv*FeCH gene [22]. The entire cDNA sequence was determined by 3′- and 5′- RACE experiments. This sequence could be mapped to the genomic DNA sequence of this organism obtained from our genome sequencing project, the details of which will be published elsewhere. The genomic and cDNA sequences of the *Sv*FeCH gene are presented in Figure 1. The length of the coding region was 1122 bp including the stop codon. There was one short (49 bp) intron. The deduced amino acid sequence had a length of 373 residues and an expected molecular mass of 43.3 kDa.

In our search for the presence of other heme biosynthesis genes, BLAST homology searches were performed against nematode genome and EST databases, using human heme biosynthesis gene sequences as queries (Tables S3 and S4). Overall, many nematodes appeared to lack all the heme biosynthesis genes, as reported for *C. elegans* [13]. However, some exceptions were also noticed, including the presence of the aminolevulinic acid dehydrogenase (ALAD) gene in several species and the uroporphyrinogen decarboxylase (UROD) gene in *Meloidogyne paranaensis*, the coproporphyrinogen oxidase (CPOX) gene in *Ancylostoma caninum,* and the FeCH gene in *B. malayi* and *Strongyloides ratti*. No heme biosynthesis gene other than FeCH was found in our *S. venezuelensis* genome and transcriptome data using the human sequences as queries. We did not obtain any significant hit from the BLAST analyses for *S. venezuelensis* genome and transcriptome datasets using the ALAD, UROD, and CPOX gene sequences identified in the nematode genome/EST datasets (see above) as queries. When the *Sv*FeCH protein sequence was used as a query for the BLAST analysis, two additional species were found to carry FeCH gene (Table S5), namely *Litomosoides sigmodontis* and *Onchocerca volvulus*. The *S. venezuelensis* sequence was also used for BLAST searches against NCBI non-redundant protein database, which led to the identification of two more nematode species that carry FeCH, namely *Dirofilaria immitis* and *Acanthocheilonema viteae*. These results are interesting because all the species found to carry the FeCH gene were animal parasites (filarial nematodes in clade III and *Strongyloides* in clade IV).

**Figure 2:**
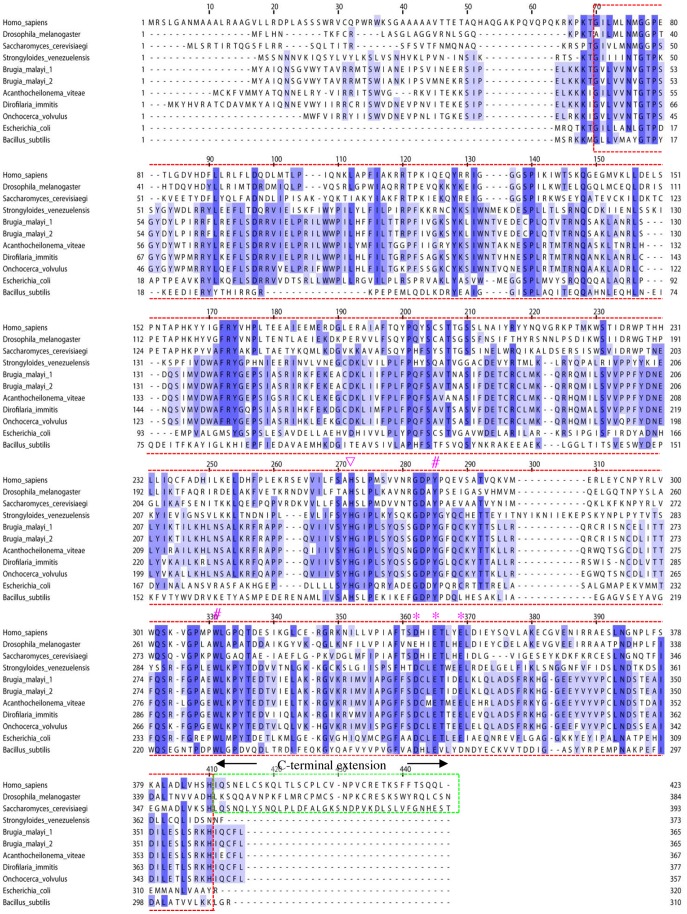
Multiple sequence alignment of FeCH sequences. The FeCH sequences were taken from the NCBI protein database together with the *S. venezuelensis* sequence (this study); *Homo sapiens* (CAB65962), *Drosophila melanogaster* (AAC26225), *Saccharomyces cerevisiae* (EDV10759), *Brugia malayi* 1 (ADI33748), *Brugia malayi* 2 (ADI33749), *Acanthocheilonema viteae* (ADI33750), *Dirofilaria immitis* (ADI33752), *Onchocerca volvulus* (ADI33751), *Escherichia coli* (AP_001124), and *Bacillus subtilis* (NP_388894). The sequences were computationally aligned by the ClustalX program [55]. The catalytic core and the C-terminal extension are boxed by red and green dotted lines, respectively. A histidine residue reported to be critical for metal substrate binding (H263, human sequence numbering) is indicated by an inverted triangle. A cluster of three acidic residues are marked with asterisks. Two residues at the active site that were reported to be identical in all known FeCH sequences (Y276 and W310) [56] are indicated by number (#) marks.

A multiple sequence alignment of FeCH protein sequences from selected organisms is presented in Figure 2. Amino acid residues in the catalytic core (boxed by a red dotted line) displayed moderate similarity. Key residues for FeCH activity, such as H263 (human sequence numbering), which was proposed to be involved in metal substrate binding [23], [44], were well conserved. Characteristically, nematode (*S. venezuelensis* and *B. malayi*) FeCH lacked a protein region called the “C-terminal extension,” a short (approximately 30–50 amino acid residues) stretch of sequences at the C-terminus of the protein that is commonly present in the FeCH of non-nematode opisthokonts [23], [45] (boxed by a green dotted line in Figure 2). To measure the similarities of these selected sequences, BLAST scores and amino acid identities were retrieved by the BLASTP program (Table S6). All the nematode FeCH sequences had higher BLAST scores and percent similarity values to the *E. coli* sequence (BLAST score: 202–221; similarity: 33.6%–36.8%) than to human (92–106 and 26.8%–27.5%, respectively), *Drosophila* (93–102 and 24.0%–25.9%, respectively) and *Saccharomyces* sequences (74–96 and 23.0%–27.2%, respectively). When BLAST homology searches were conducted using the *Sv*FeCH protein sequence as a query against the NCBI non-redundant protein as described above, virtually all the top hits were bacterial sequences excluding the sequences of filarial nematodes (data not shown). These findings prompted us to conduct a phylogenetic analysis to better clarify the evolutionary origin of nematode FeCH genes.

### Phylogenetic Analysis

**Figure 3:**
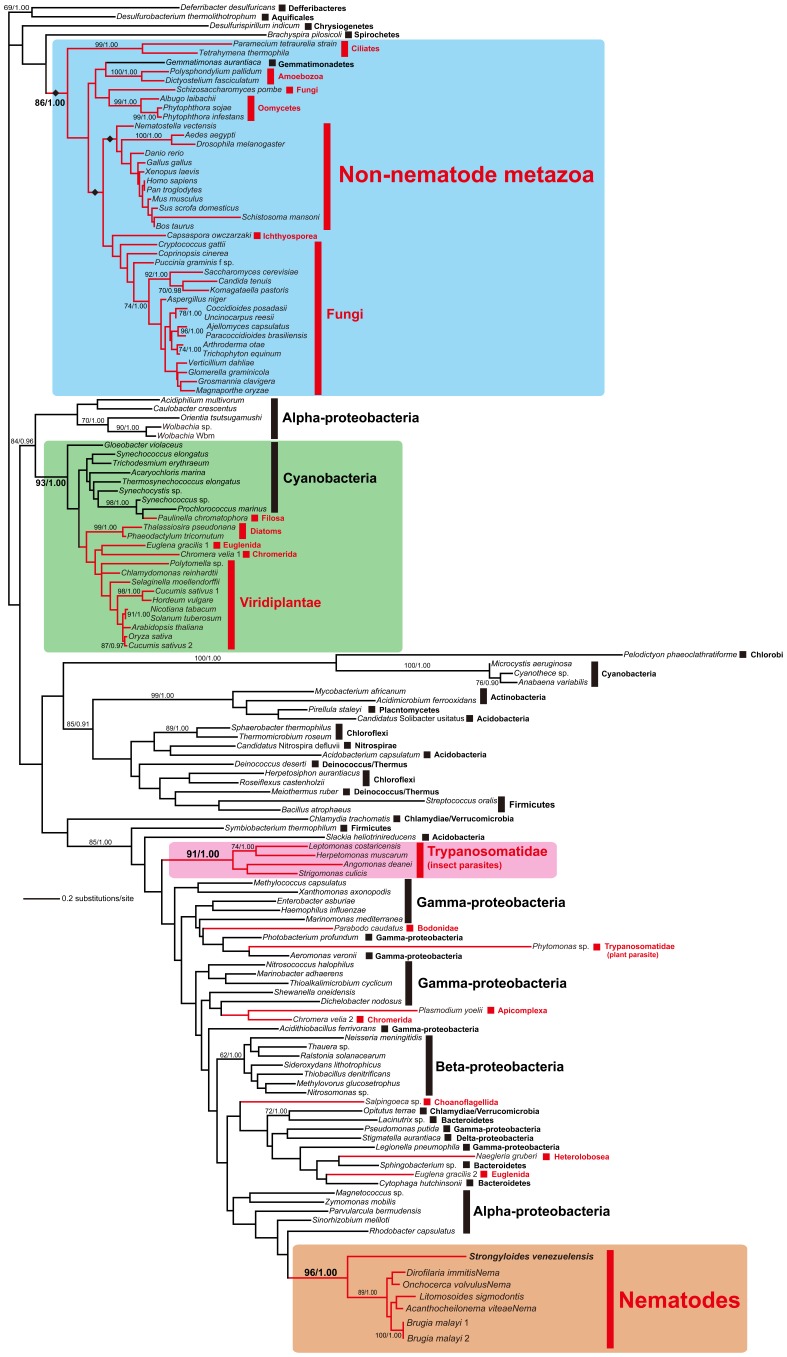
Phylogenetic analysis of FeCH sequences. The ML phylogeny inferred from FeCH amino acid sequences from 71 bacteria and 65 eukaryotes. Numerical values at the nodes represent MLBPs and BPPs. Only MLBPs greater than 60% are shown. The majority of the FeCH homologs sampled from eukaryotes (colored in red) were separated into four clades shaded in blue, green, pink, and orange. The homologs from *Strongyloides venezuelensis* identified in this study formed the ‘orange’ clade with those of other nematodes. We compared the ML tree and the alternative hypotheses for the origin of the nematode FeCH genes by pruning and regrafting the entire nematode clade (shaded in orange) to the branches marked by diamonds in the ‘blue’ clade.

The amino acid alignment of FeCH sampled from 71 eukaryotic and 71 bacterial species was phylogenetically analyzed by ML and Bayesian methods (Figure 3). Overall, the FeCH trees inferred by the ML and Bayesian methods were concordant with each other as well as the results of previously published FeCH phylogenies [7], [46], [47], [48]. Four major clades including the FeCH homologues sampled from eukaryotes were reconstructed with ML bootstrap support values (MLBPs) of 86%–96% and a Bayesian posterior probability (BPP) of 1.00 (shaded in blue, green, pink, and orange; Figure 3): (1) a ‘blue’ clade comprising a single bacterial homolog (*Gemmatimonas aurantiaca*) and those of eukaryotes–non-nematode metazoans, fungi, *Capsaspora owczarzaki*, oomycetes, amoebozoans, and ciliates; (2) a ‘green’ clade of the homolog of cyanobacteria including an obligate endosymbiont in the testate amoeba, *Paulinella chromatophora* [49], and putative plastid homolog in photosynthetic eukaryotes; (3) a ‘pink’ clade comprising the homolog of insect trypanosomatids [3]; (4) an ‘orange’ clade comprising the homolog of parasitic nematodes including *S. venezuelensis*. Other homologs sampled from eukaryotes were scattered amongst the bacterial homologues, and they exhibited no specific evolutionary affinity to other homologs.

The FeCH phylogeny suggested that the homologs from non-nematode metazoans nested in the ‘blue’ clade and those of nematodes forming the ‘orange’ clade were distantly related to each other. Although they received little support from the ML bootstrap and Bayesian analyses, the homologs from non-nematode metazoans, *Capsaspora*, and fungi were grouped together, corresponding to members of Opisthokonta, a well-established monophyletic assemblage [50]. Curiously, the nematode FeCH homologs formed a robust clade with an MLBP of 96% and BPP of 1.00, being distinct from other metazoan homologs. This tree topology can be rationalized by the vertical inheritance of FeCH genes from the ancestral opisthokont species to non-nematode metazoans and horizontal transfer of a FeCH gene between the ancestral nematodes and a non-metazoan organism. This conjecture was further supported by a topology test comparing the ML tree shown in Figure 3 with three alternative trees, in which the nematode homologs were enforced to branch at the base of (1) the non-nematode metazoan clade, (2) the clade of the opisthokont homologues (excluding that of *Schizosaccharomyces pombe*), and (3) the ‘blue’ clade composed of the eukaryotic and *Gemmatimonas* homologs (highlighted by diamonds in Figure 3). Importantly, all the alternative trees were successfully rejected with very small *p* values (2.0×10^−78^–2.0×10^−36^).

### Chelatase Assay using Recombinant *Sv*FeCH

**Figure 4:**
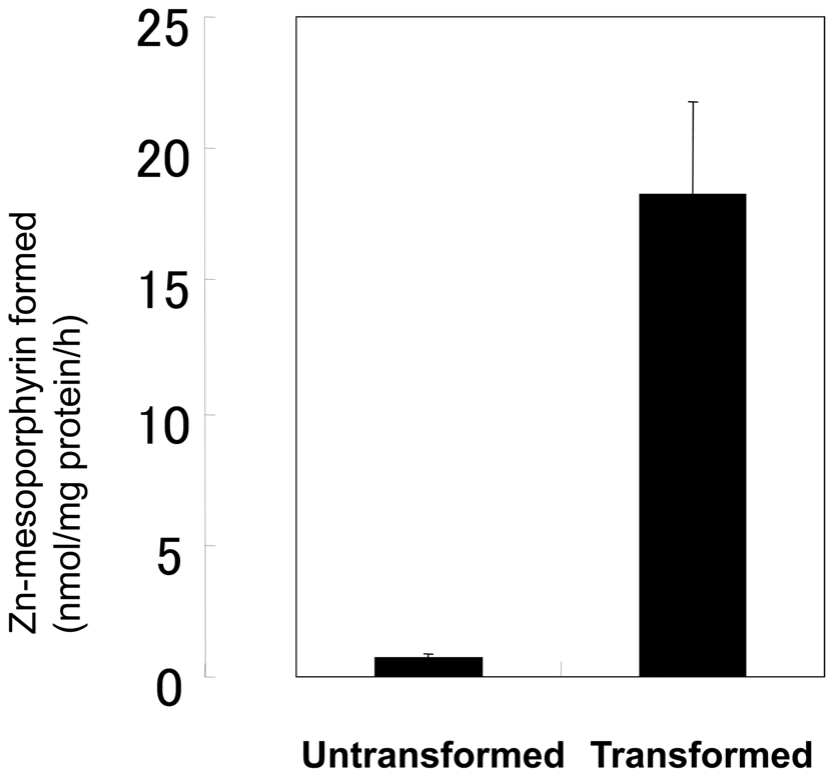
Chelatase assay using bacterially expressed recombinant *Sv*FeCH. The cell extracts were incubated with 20 mM Tris-HCl, pH 8.0, 0.1% Tween 20, 15 µM mesoporphyrin IX, and 40 µM zinc acetate in a final volume of 200 µl at 30°C for 60 min. The formation of zinc mesoporphyrin was measured. Data are expressed as the mean ± SD of triplicate experiments.

To determine whether the FeCH gene of *S. venezuelensis* identified in the present study encodes an active enzyme, we conducted a chelatase assay using a bacterially expressed recombinant *Sv*FeCH. We constructed an expression plasmid, pET-*Sv*FeCH, which was used to transform *E. coli* strain BL21. Protein expression was induced by incubation with 0.3 mM IPTG at 30°C for 2 h. The enzyme activity was measured using the cell extracts of untransformed and transformed bacteria. The FeCH activity in transformed bacteria, which was derived from overexpressed *Sv*FeCH and endogenous *E. Coli* FeCH, was much higher than that in the untransformed control, which originated solely from endogenous FeCH, indicating that the enzyme was active (Figure. 4).

### Genetic Complementation Assay of hemH Deficient *E. coli*

**Figure 5:**
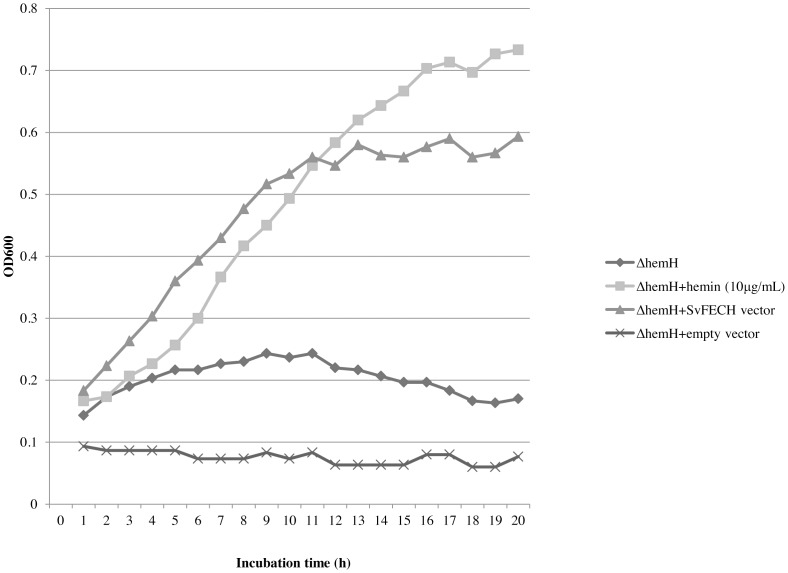
Genetic complementation assay of ΔhemH *E. coli*. An untransformed ΔhemH strain of *E. coli* was grown in the absence (diamond) or presence 10 µg/ml hemin (square). In the same experiment, a transformed ΔhemH strain of *E. coli* either with *Sv*FeCH gene expression vector (triangle) or with empty vector (x-mark) was cultured in the absence of hemin. OD_600_ was measured every hour up to 20 h to monitor bacterial growth.

The VS200 strain of *E. coli* K12, a hemH null-mutant, was used for the gene complementation assay, and the results are shown in Figure 5. VS200 could not grow in LB medium, unless hemin (10 µg/ml) was supplemented. The expression of *Sv*FeCH by pFLAG-CTC-*Sv*FeCH made the bacteria capable of growing in the LB medium in the absence of hemin. Transforming the bacteria with the control vector (pFLAG-CTC) did not have such an effect. Therefore, it was concluded that *Sv*FeCH is an active enzyme that can function as FeCH.

### Expression of FeCH during the Life Cycle of *S. venezuelensis*

**Figure 6:**
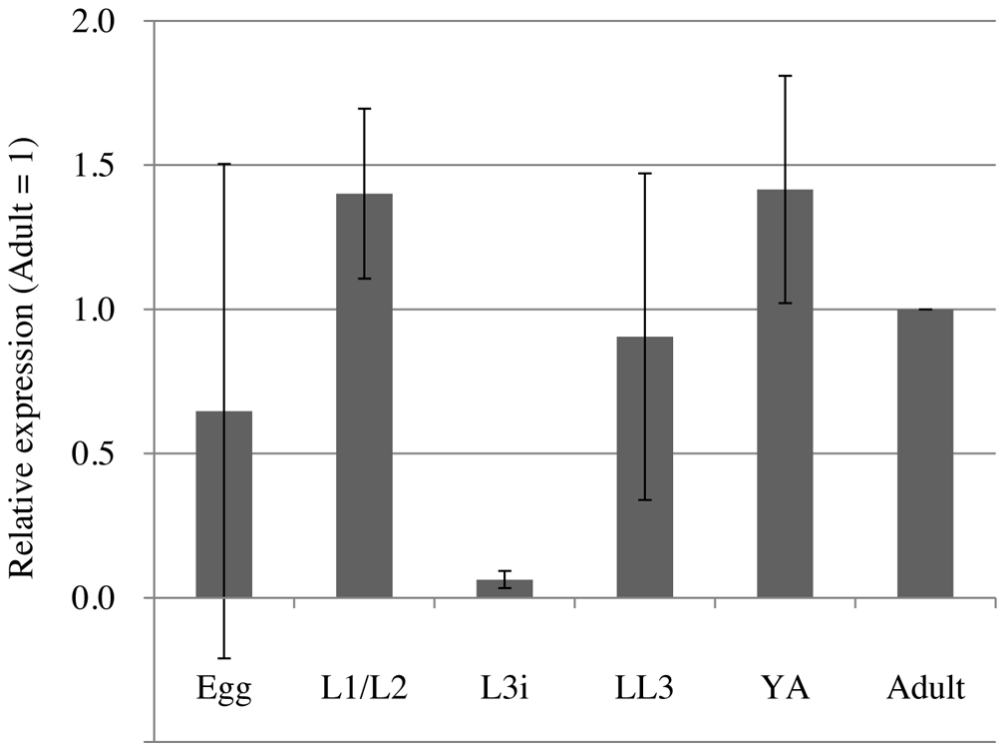
Expression analysis of *Sv*FeCH gene by real-time RT-PCR. mRNA abundance is shown ***relative*** to the expression level at the adult stage, after normalizing to 18S rRNA expression levels. The bars represent the means and standard deviations (±) of biological triplicates. Real-time RT-PCR was performed in triplicate wells for each biological replicate.

The relative expression levels of *Sv*FeCH mRNA were assessed by real-time RT-PCR analysis using RNA samples prepared from the six major developmental stages of *S. venezuelensis* (Figure 6). It was observed that although *Sv*FeCH mRNA expression was present throughout the stages, it was relatively low in L3i.

## Discussion

We demonstrated that a gene for FeCH exists in the *S. venezuelensis* genome. Although the presence of the FeCH gene in the draft genome of *B. malayi* was reported previously [24], [25], no further characterization was reported. The present study represents the first cloning and characterization of nematode FeCH, particularly in an evolutionary context.

Phylogenetic analyses revealed that nematode FeCH forms a distinct clade from that of non-nematode metazoans, indicating that the evolutionary origin of nematode FeCH is fundamentally different from that of the FeCH genes of other metazoan organisms. In the ML phylogeny, the nematode clade was placed within the homologs from a subset of alpha-proteobacteria, although the statistical support for this hypothesis is inconclusive. If the affinity between the nematode and alpha-proteobacterial FeCH homologs is genuine, then an as-yet-unknown alpha-proteobacterium was the source of the FeCH homologs working in the extant nematodes. This hypothesis is intriguing because replacement of the eukaryotic FeCH gene by a bacterial FeCH gene had been suggested only for unicellular eukaryotes, such as apicomplexan parasites (*Plasmodium falciparum, P. chabaudi, P. berghei, Eimeria tenella, Toxoplasma gondii,* and *Neospora caninum*) [47], [48], [51], the chromerid *Chromera velia* [47], rhodophytes (*Cyanidioschyzon merolae*, *Porphyra yezoensis*, and *Galdieria sulphuraria*) [48], and the euglenid *Euglena gracilis* [46].

BLAST analysis of the sequenced nematode genomes and transcriptomes revealed that the FeCH gene is present only in *Strongyloides* (clade IV) and filarial parasites (clade III). It is still not clear at which point of nematode evolution the proposed horizontal gene transfer event occurred. Regarding *B. malayi* and related filarial nematodes, horizontal gene transfer from *Wolbachia*, a bacterial symbiont, is known to have occurred [52]. However, the FeCH sequences present in nematode genomes do not appear to originate from *Wolbachia* based on the positions of the *Wolbachia* species in the phylogenetic tree (Figure 3).

**Figure 7:**
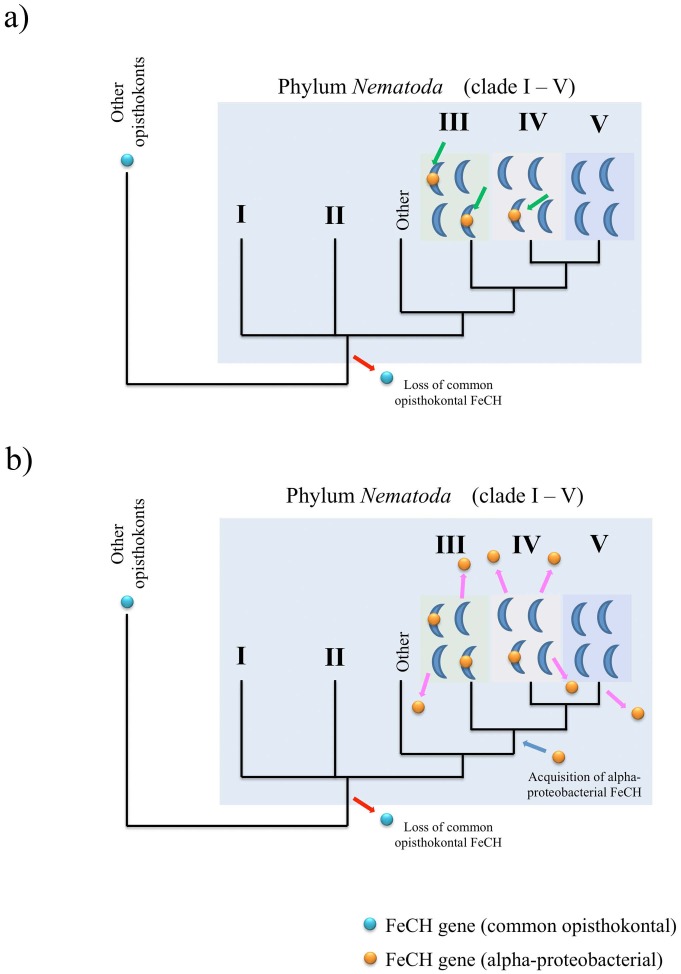
Proposed hypotheses for the loss of the original (common opisthokontal) FeCH gene and the re-acquisition of alpha-proteobacterial FeCH in the evolution of the phylum Nematoda. The initial loss of the common opisthokontal FeCH gene may have occurred at the common ancestor level (red arrows). (a) Scenario 1: The first scenario hypothesizes that alpha-proteobacterial FeCH was acquired independently by some species in clades III and IV (green arrows). b) Scenario 2: Reacquisition of FeCH from an alpha-proteobacterium may have occurred at the common ancestor level of clades III, IV and V (blue arrow) followed by a secondary loss in some species in clade III and IV and in the branch leading to clade V (pink arrows). The phylogenetic relationships of the nematode clades are based on Sommer and Streit [10].

We hypothesize two possible scenarios concerning the evolutionary histories of FeCH genes in nematodes, using a current view of the phylogenetic relationship of nematode clades [10]. Because no nematode species possesses the ‘blue clade’ FeCH commonly found in opisthokonts, it can be speculated that this type of FeCH was lost early in nematode evolution (Figure. 7). *Strongyloides* and the filarias may have acquired FeCH genes from alpha-proteobacteria independently. Alternatively, a common ancestral lineage leading to clades III, IV, and V may have received such an alpha-proteobacterial FeCH gene (scenarios 1 and 2, respectively: Fig. 7a and 7b). For scenario 1 to be true, the hypothetical alpha-proteobacterial species that provided FeCH genes to *Strongyloides* and filarias, need to be closely related to each other, because the nematode homologs were robustly grouped together in the FeCH phylogeny (Figure. 3). In scenario 2, the lateral transfer of a bacterial FeCH gene occurred through an ancestor leading to species that belong to clades III, IV, and V, and again, the FeCH gene disappeared in some species in clades III and IV such as *Ascaris* and *Meloidogyne* and in the branch leading to clade V (Figure. 7b).

Among the parasitic nematodes, the reason why only *Strongyloides* and filarias needed to reacquire (scenario 1) or retain (scenario 2) FeCH gene is unclear, particularly when the other six heme biosynthesis genes are still absent. This situation (the presence of FeCH gene in the absence of other heme biosynthesis genes) has been documented for a limited number of organisms, such as *Haemophilus influenzae* [53] and *P. serpens* [9]. As was suggested for *H. influenzae* [9], [54], there may be a possibility that FeCH is used to obtain Fe^2+^ through its reverse activity rather than obtain heme from protoporphyrin IX using its forward activity.

## Supporting Information

Table S1 **List of primers used in this study.**
(PDF)

Table S2 **Taxonomic affiliation and accession numbers for the sequences considered in the phylogenetic analysis.**
(PDF)

Table S3 **BLAST homology search against predicted proteins from nematode genome projects.**
(PDF)

Table S4 **BLAST homology search against nematode EST database (NEMBASE 4).**
(PDF)

Table S5 **BLAST homology search against nematode EST database (NEMBASE 4) using** ***Strongyloides venezuelensis*** **ferrochelatase sequence as a query.**
(PDF)

Table S6 **Sequence similarities of FeCH proteins assessed by BLAST program.**
(PDF)
