# Supplementary material for: Identification of a Bacteria-Like Ferrochelatase in Strongyloides venezuelensis, an Animal Parasitic Nematode
Source: PLoS One. 2013 Mar 13;8(3):e58458. doi: 10.1371/journal.pone.0058458 (PMC3596385; doi:10.1371/journal.pone.0058458)
Supplement: Table S1 — List of primers used in this study. (PDF) [file pone.0058458.s001.pdf]

Table S1 List of primers used in this study

Number	Name	5' -> 3' sequence	direction	Restriction sequence
1	ENM059	TGTTGGTGGAGCATGTGATG	forward	-
2	ENM008	GACCACGCGTATCGATGTCGAC	-	-
3	ENM060	CCCACTCCCATATACCGTGA	forward	-
4	ENM070	TCCTCAATATTGTGTGGTCCAT	reverse	-
5	ENM072	TGGTGATTCATCCTTCTCCAT	reverse	-
6	ENM073	ATGTCGTCTAACAATAATGTTAAAATTC	forward	-
7	ENM5_6_7	GACCACGCGTATCGATGTCGACTTTTTTTTTTTTTTTTV	-	-
8	ENM008	GACCACGCGTATCGATGTCGAC	-	-
9	ENM074	TTATTTTGTATTTATATTAATTAACTTTTCAAG	reverse	-
10	TKT001	AAAGAATTCGAATATTAATAGTATCAA	forward	EcoRI
11	TKT002	AAAAAGCTTCTAAAAGTTATTACTAT	reverse	HindIII
12	ENM089	ATCGCTCGAGATGTCGTCTAACAATAATGTTAAAATTCAATC	forward	XhoI
13	ENM098	GCTAAGATCTCTAAAAGTTATTACTATCAATTAATTGACATAGAA	reverse	BglII
14	ENM056	CCTCAATATTGTGTGGTCCATATC	reverse	-
15	ENM057	TGGAGAAGGATGAATCACCATTATTA	forward	-
16	377F	AAGGAAAGGGCAAGTCTGGT	forward	-
17	501R	CATCCAAGATGCTCATTACACA	reverse	-
