# Supplementary material for: Identification of a Bacteria-Like Ferrochelatase in Strongyloides venezuelensis, an Animal Parasitic Nematode
Source: PLoS One. 2013 Mar 13;8(3):e58458. doi: 10.1371/journal.pone.0058458 (PMC3596385; doi:10.1371/journal.pone.0058458)
Supplement: Table S2 — Taxonomic affiliation and accession numbers for the sequences considered in the phylogenetic analysis. (PDF) [file pone.0058458.s002.pdf]

**Table S2** Taxonomic affiliation and accsession numbers for the sequences considered in the phylogene

| Higher Taxon | Accession | Organism names |
|---|---|---|
| Alpha-proteobacteria | YP004285020 | Acidiphilium multivorum AIU301 |
| | NP422556 | Caulobacter crescentus CB15 |
| | YP001937208 | Orientia tsutsugamushi str Ikeda |
| | YP003854109 | Parvularcula bermudensis HTCC2503 |
| | YP004550179 | Sinorhizobium meliloti AK83 |
| | YP003576367 | Rhodobacter capsulatus SB 1003 |
| | YP162038 | Zymomonas mobilis subsp. mobilis ZM4 |
| | YP865616 | Magnetococcus sp. MC-1 |
| | YP865616 | Wolbachia sp. wRi |
| | YP198549 | Wolbachia Wbm |
| Beta-proteobacteria | YP003082820 | Neisseria meningitidis alpha14 |
| | YP002355372 | Thauera sp. MZ1T |
| | YP003523671 | Sideroxydans lithotrophicus ES-1 |
| | YP315791 | Thiobacillus denitrificans ATCC 25259 |
| | YP003051656 | Methylovorus glucosetrophus SIP3-4 |
| | YP004294842 | Nitrosomonas sp. AL212 |
| | YP003751536 | Ralstonia solanacearum PSI07 |
| Gamma-proteobacteria | YP003526615 | Nitrosococcus halophilus Nc4 |
| | YP004135566 | Haemophilus influenzae F3031 |
| | YP004827520 | Enterobacter asburiae LF7a |
| | YP004392921 | Aeromonas veronii B565 |
| | YP129238 | Photobacterium profundum SS9 |
| | YP004313230 | Marinomonas mediterranea MMB-1 |
| | YP004785265 | Acidithiobacillus ferrivorans SS3 |
| | YP001266120 | Pseudomonas putida F1 |
| | YP003617676 | Legionella pneumophila 2300/99 Alcoy |
| | YP001210125 | Dichelobacter nodosus VCS1703A |
| | YP004537681 | Thioalkalimicrobium cyclicum ALM1 |
| | NP718902 | Shewanella oneidensis MR-1 |
| | YP114114 | Methylococcus capsulatus str. Bath |
| | YP004853627 | Xanthomonas axonopodis pv citrumelo F1 |
| | YP005885474 | Marinobacter adhaerens HP15 |
| Delta-proteobacteria | YP003951943 | Stigmatella aurantiaca DW4/3-1 |
| Chlamydiae/Verrucomicro | YP001819352 | Opitutus terrae PB90-1 |
| | NP219999 | Chlamydia trachomatis D/UW-3/CX |
| Bacteroidetes | YP 680066 | Cytophaga hutchinsonii ATCC 33406 |
| | YP 004316311 | Sphingobacterium sp. 21 |
| | YP 004579396 | Lacinutrix sp. 5H-3-7-4 |
| Actinobacteria | YP 003145049 | Slackia heliotrinireducens DSM 20476 |
| | YP 004723195 | Mycobacterium africanum GM041182 |
| | YP 003110115 | Acidimicrobium ferrooxidans DSM 10331 |
| Chloroflexi | YP 002522588 | Thermomicrobium roseum DSM 5159 |
| | YP 003320569 | Sphaerobacter thermophilus DSM 20745 |
| | YP 001432135 | Roseiflexus castenholzii DSM 13941 |
| | YP 001546311 | Herpetosiphon aurantiacus DSM 785 |
| Firmicutes | YP 073989 | Symbiobacterium thermophilum IAM 14863 |
| | YP 004326063 | Streptococcus oralis Uo5 |
| | YP 003972425 | Bacillus atrophaeus 1942 |
| Deinococcus-Thermus | YP 002785119 | Deinococcus deserti VCD115 |
| | YP 003507187 | Meiothermus ruber DSM 1279 |
| Cyanobacteria | YP 001658460 | Microcystis aeruginosa NIES-843 |
| | YP 323262 | Anabaena variabilis ATCC 29413 |
| | YP 003137494 | Cyanothece sp. PCC 8802 |
| | NP 442453 | Synechocystis sp. PCC 6803 |
| | NP 923785 | Gloeobacter violaceus PCC 7421 |
| | YP 723781 | Trichodesmium erythraeum IMS101 |
| | YP 172078 | Synechococcus elongatus PCC 6301 |
| | YP 001550413 | Prochlorococcus marinus str MIT 9211 |
| | YP 001516290 | Acaryochloris marina MBIC11017 |
| | NP 683006 | Thermosynechococcus elongatus BP-1 |
| | YP 731203 | Synechococcus sp. CC9311 |
| Acidobacteria | YP 002755471 | Acidobacterium capsulatum ATCC 51196 |
| | YP 822312 | Candidatus Solibacter usitatus Ellin6076 |
| Spirochetes | YP 003786643 | Brachyspira pilosicoli 95/1000 |
| Chrysiogenetes | YP 004112072 | Desulfurispirillum indicum S5 |
| Gemmatimonadetes | YP 002762295 | Gemmatimonas aurantiaca T-27 |
| Deferribacteres | YP 003495576 | Deferribacter desulfuricans SSM1 |
| Aquificales | YP 004281002 | Desulfurobacterium thermolithotrophum DSM |
| Chlorobi | YP 002018719 | Pelodictyon phaeoclathratiforme BU-1 |
| Nitrospirae | YP 003796898 | Candidatus Nitrospira defluvii |
| Planctomycetes | YP 003370976 | Pirellula staleyi DSM 6068 |

**Table S2 (continued)**

| Higher Taxon | Accession | Organism names |
|---|---|---|
| Metazoa | EAT43108 | Aedes aegypti |
| | AAC26225 | Drosophila melanogaster |
| | AF2503681 | Danio rerio |
| | AAB94626 | Xenopus laevis |
| | CAB65962 | Homo sapiens |
| | AAZ78230 | Pan troglodytes |
| | AAA79169 | Bos taurus |
| | BAI47962 | Sus scrofa domesticus |
| | AAA37615 | Mus musculus |
| | AAB66503 | Gallus gallus |
| | CCD75202 | Schistosoma mansoni |
| | EDO40867 | Nematostella vectensis |
| Fungi | NP587720 | Schizosaccharomyces pombe 972h- |
| | XP003194818 | Cryptococcus gattii WM276 |
| | EFP82759 | Puccinia graminis f.sp. tritici CRL 75-36-700-3 |
| | AAA34667 | Saccharomyces cerevisiae |
| | CCA39904 | Komagataella pastoris CBS 7435 |
| | EGV64906 | Candida tenuis ATCC 10573 |
| | XP001396807 | Aspergillus niger CBS 513.88 |
| | EFW23046 | Coccidioides posadasii str. Silveira |
| | XP002544726 | Uncinocarpus reesii 1704 |
| | EEH10395 | Ajellomyces capsulatus G186AR |
| | EEH17729 | Paracoccidioides brasiliensis Pb03 |
| | EEQ29549 | Arthroderma otae CBS 113480 |
| | EGE07189 | Trichophyton equinum CBS 127.97 |
| | EGY20619 | Verticillium dahliae VdLs.17 |
| | EFQ30694 | Glomerella graminicola M1.001 |
| | EHA54679 | Magnaporthe oryzae 70-15 |
| | EFX05816 | Grosmannia clavigera kw1407 |
| | EAU91411 | Coprinopsis cinerea okayama7#130 |
| Amoebozoa | EGG22223 | Dictyostelium fasciculatum |
| | EFA74915 | Polysphondylium pallidum PN500 |
| Ciliates | XP001347260 | Paramecium tetraurelia strain d4-2 |
| | XP001017797 | Tetrahymena thermophila |
| Kinetoplastida | AEM25314 | Phytomonas sp. JMPA-2011 |
| | AEM25309 | Angomonas deanei |
| | AEM25307 | Strigomonas culicis |
| | AEM25313 | Herpetomonas muscarum |
| | AEM25312 | Leptomonas costaricensis |
| | AEM25316 | Parabodo caudatus |
| Euglenida | AEA86537 | Euglena gracilis |
| | AEA86538 | Euglena gracilis |
| Chromerida | AEQ18828 | Chromera velia |
| | AEQ18827 | Chromera velia |
| Stramenopiles | XP 002286994 | Thalassiosira pseudonana CCMP1335 |
| | XP 002179541 | Phaeodactylum tricornutum CCAP 1055 |
| | CCA26575 | Albugo laibachii Nc14 |
| | XP 002899013 | Phytophthora infestans T30-4 |
| | EGZ14268 | Phytophthora sojae |
| Choanoflagellida | EGD74306 | Salpingoeca sp. ATCC 50818 |
| Heterolobosea | XP 002681413 | Naegleria gruberi |
| Apicomplexa | CAD12105 | Plasmodium yoelii |
| Chlorophyta | EDP03868 | Chlamydomonas reinhardtii |
| | AF3329631 | Polytomella sp. Pringsheim 198.80 |
| Streptophyta | EFJ10716 | Selaginella moellendorffii |
| | BAB20760 | Cucumis sativus |
| | BAA22284 | Oryza sativa |
| | CAA73614 | Arabidopsis thaliana |
| | CAC50871 | Nicotiana tabacum |
| | CAA06705 | Solanum tuberosum |
| | AAB71887 | Hordeum vulgare |
| | BAA05102 | Cucumis sativus |
| Ichthyosporea | EFW39873 | Capsaspora owczarzaki ATCC 30864 |
| Rhizaria | ACB43300 | Paulinella chromatophora |
| Nematodes | ADI33752 | Dirofilaria immitis |
| | ADI33751 | Onchocerca volvulus |
| | ADI33750 | Acanthocheilonema viteae |
| | ADI33748 | Brugia malayi |
| | ADI33749 | Brugia malayi |
| | AB710465* | Strongyloides venezuelensis |
| | LSC01092_1** | Litomosoides sigmodontis |

Accession numbers are for NCBI GenBank, unless specified
*Identified in this study
** Obtained from NEMBASE4 (http://www.nematodes.org/nembase4/)
