# Supplementary material for: Identification of a Bacteria-Like Ferrochelatase in Strongyloides venezuelensis, an Animal Parasitic Nematode
Source: PLoS One. 2013 Mar 13;8(3):e58458. doi: 10.1371/journal.pone.0058458 (PMC3596385; doi:10.1371/journal.pone.0058458)
Supplement: Table S3 — BLAST homology search against predicted proteins from nematode genome projects. (PDF) [file pone.0058458.s003.pdf]

Table S3 BLAST homologiy search against predicted proteins from published nematode genome projects

nematode clade	Species	Mode of life	ALAD	PBGD	UROS	UROD	CPOX	PPOX	FECH
V	*C. briggsae*	FL	-	-	-	-	-	-	-
V	*C. elegans*	FL	-	-	-	-	-	-	-
V	*P. pacificus*	FL	2×10^{-16}	-	-	-	-	-	-
IV	*M. incognita*	PP	-	-	-	-	-	-	-
IV	*M. hapla*	PP	-	-	-	-	-	-	-
IV	*B. xylophilus*	PP	-	-	-	-	-	-	-
III	*B. malayi*	AP	-	-	-	-	-	-	6×10^{-21}
III	*A. suum*	AP	-	-	-	-	-	-	-
I	*T. spiralis*	AP	-	-	-	-	-	-	-
	D. malanogaster	FL	3×10^{-19}	8×10^{-78}	1×10^{-25}	1×10^{-105}	1×10^{-123}	9×10^{-79}	1×10^{-111}

Human sequences of heme biosynthetic genes used as queries are as follows: ALAD (aminolevulinic acid dehydratase, AY319481.1) PBGD (porphobilinogen deaminase, NM_000190.3), UROS (uroporphyrinogen III synthase, BC002573.2), UROD (uroporphyrinogen decarboxylase, CR456976.1), CPOX (coproporphyrinogen oxidase, NM_000097.5), PPOX (protoporphyrinogen oxidase, NM_000309.3), FECH (ferrochelatase, BC039841.1). A cut-off E-value of 1×10^{-4} was used in this BLASTx analysis. Minus mark indicates no hits were found. In case hits were found, E-values for respective hits are shown. FL: free-living, PP: plant-parasitic, AP: animal-parasitic.
