# Supplementary material for: Identification of a Bacteria-Like Ferrochelatase in Strongyloides venezuelensis, an Animal Parasitic Nematode
Source: PLoS One. 2013 Mar 13;8(3):e58458. doi: 10.1371/journal.pone.0058458 (PMC3596385; doi:10.1371/journal.pone.0058458)
Supplement: Table S4 — BLAST homology search against nematode EST database (NEMBASE 4). (PDF) [file pone.0058458.s004.pdf]

Table S4 Blast homology search against nematode EST database (NEMBASE 4)

Gene	NEMBASE4 ID	species	clade	mode of life	E-value
ALAD	ACC10593_1	*Ancylostoma caninum*	V	AP	4 x 10-^{26}
ALAD	HBC00099_1	*Heterorhabditis bacteriophora*	V	EP	2 x 10^{-5}
ALAD	MHC08010_1	*Meloidogyne hapla*	IV	PP	6 x 10^{-39}
ALAD	NAC01466_1	*Necator americanus*	V	AP	9 x 10^{-22}
ALAD	OVC00266_1	*Onchocerca volvulus*	III	AP	6 x 10^{-5}
ALAD	WBC01431_1	*Wuchereria bancrofti*	III	AP	2 x 10^{-39}
UROD	MPC01279_1	*Meloidogyne paranaensis*	IV	PP	2 x 10^{-23}
CPOX	ACC18701_1	*Ancylostoma caninum*	V	AP	9 x 10^{-21}
FECH	SRC06613_1	*Strongyloides ratti*	IV	AP	7 x 10^{-17}

Human sequences of heme biosynthetic genes used as queries are as follows: ALAD (aminolevulinic acid dehydratase, AY319481.1) PBGD (porphobilinogen deaminase, NM_000190.3), UROS (uroporphyrinogen III synthase, BC002573.2), UROD (uroporphyrinogen decarboxylase, CR456976.1), CPOX (coproporphyrinogen oxidase, NM_000097.5), PPOX (protoporphyrinogen oxidase, NM_000309.3), FECH (ferrochelatase, BC039841.1). A cut-off E-value of 1 x 10^{-4} was used in this tBLASTx analysis. Minus mark indicates no hits were found. In case hits were found, E-values for respective hits are shown. PP: plant-parasitic, AP: animal-parasitic, EP: entomopathogenic.
