# Supplementary material for: Identification of a Bacteria-Like Ferrochelatase in Strongyloides venezuelensis, an Animal Parasitic Nematode
Source: PLoS One. 2013 Mar 13;8(3):e58458. doi: 10.1371/journal.pone.0058458 (PMC3596385; doi:10.1371/journal.pone.0058458)
Supplement: Table S5 — BLAST homology search against nematode EST database (NEMBASE 4) using Strongyloides venezuelensis ferrochelatase sequence as a query. (PDF) [file pone.0058458.s005.pdf]

Table S5 BLAST homology search against nematode EST database (NEMBASE4) using *Strongyloides venezuelensis* ferrochelatase sequence as a query

NEMBASE4 ID	species	clade	mode of life	E-value
SRC06613_1	*Strongyloides ratti*	IV	AP	1×10^{-177}
LSC01092_1	*Litomosoides sigmodontis*	III	AP	1×10^{-47}
OVC00158_1	*Onchocerca volvulus*	III	AP	1×10^{-10}

S. venezuelensis ferrochelatase protein sequence was used as a query against NEMBASE4 using tBLASTn algorithm (E-value cut-off, 1×10^{-4}). AP: animal parasitic.
