# Supplementary material for: Identification of a Bacteria-Like Ferrochelatase in Strongyloides venezuelensis, an Animal Parasitic Nematode
Source: PLoS One. 2013 Mar 13;8(3):e58458. doi: 10.1371/journal.pone.0058458 (PMC3596385; doi:10.1371/journal.pone.0058458)
Supplement: Table S6 — Sequence similarities of FeCH proteins assessed by BLAST program. (PDF) [file pone.0058458.s006.pdf]

Table S6 Sequence similarities of FeCH proteins assessed by BLAST program

	Homo sapiens	Drosophila melanogaster	Saccharomyces cerevisiae	Strongyloides venezuelensis	Brugia malayi 1	Brugia malayi 2	Acanthocheilonema viteae	Dirofilaria immitis	Onchocerca volvulus	Escherichia coli	Bacillus subtilis
Homo sapiens	885 (100)										
Drosophila melanogaster	431(55.7)	808 (100)									
Saccharomyces cerevisiae	364 (51.3)	296 (42.5)	821 (100)								
Strongyloides venezuelensis	107 (25.8)	67.8 (24.1)	84 (24.0)	758 (100)							
Brugia malayi 1	103 (27.2)	102 (25.3)	84 (24.0)	292 (45.0)	755 (100)						
Brugia malayi 2	106 (27.5)	102 (24.9)	84 (24.0)	291 (45.0)	749 (98.9)	756 (100)					
Acanthocheilonema viteae	92 (25.4)	93 (24.0)	74 (23.0)	296 (45.6)	615 (80.3)	619 (80.8)	764 (100)				
Dirofilaria immitis	100 (27.0)	102 (25.2)	84 (24.1)	312 (46.7)	626 (81.0)	627 (81.0)	619 (79.5)	789 (100)			
Onchocerca volvulus	98 (26.8)	95 (24.6)	83 (23.8)	305 (46.5)	606 (79.0)	607 (79.0)	608 (81.1)	662 (86.3)	743 (100)		
Escherichia coli	105（27.0）	101 (25.9)	96 (27.2)	202 (33.6)	221 (36.8)	221 (36.8)	212 (34.9)	214 (35.5)	216 (35.5)	662 (100)	
Bacillus subtilis	127 (28.5)	129 (27.6)	99 (25.1)	42 (23.4)	59 (21.1)	57 (20.8)	64 (22.2)	64 (22.0)	64 (23.0)	77 (26.4)	640 (100)

BLAST scores and percent identity values (in brackets) were obtained by BLASTP program using the following FeCH protein sequences: *Homo sapiens* (CAB65962), *Drosophila melanogaster* (AAC26225), *Saccharomyces cerevisiae* (EDV10759), *Strongyloides venezuelensis* (BAM62638.1, this study), *Brugia malayi* 1 (ADI33748), *Brugia malayi* 2 (ADI33749), *Acanthocheilonema viteae* (ADI33750), *Dirofilaria immitis* (ADI33752), *Onchocerca volvulus* (ADI33751), *Escherichia coli* (AP_001124), and *Bacillus subtilis* (NP_388894).
